# Ruptured Sinus of Valsalva Aneurysm With 2 Simultaneous Fistulas to the Left Ventricular Outflow Tract

**DOI:** 10.1016/j.jaccas.2025.106612

**Published:** 2026-01-21

**Authors:** Ryoto Mitsumizo, Ryuichi Matsukawa, Takayuki Uchida, Jun-ichiro Nishi

**Affiliations:** a Clinical Training Center, Aso Iizuka Hospital, Iizuka, Japan; b Department of Cardiology, Aso Iizuka Hospital, Iizuka, Japan; c Department of Cardiovascular, Aso Iizuka Hospital, Iizuka, Japan

**Keywords:** aortic dissection, fistula, left-to-left shunt, multimodality imaging, Sinus of Valsalva aneurysm

## Abstract

**Background:**

Sinus of Valsalva aneurysm (SOVA) rupture usually forms fistulas into right heart chambers; left-sided ruptures are exceedingly rare.

**Case Summary:**

A 73-year-old woman presented for evaluation of ST-segment depression in V5 and V6 and moderate aortic regurgitation. Imaging revealed a noncoronary cusp–origin SOVA with 2 simultaneous fistulas into the left ventricular outflow tract. She remained asymptomatic with stable hemodynamics, allowing elective surgical repair with patch closure, direct suture, and bioprosthetic valve replacement.

**Discussion:**

The left-to-left shunt decompressed the aneurysmal sac, preventing acute heart failure. Unlike typical left-to-right ruptures, these favorable flow dynamics allowed safe delayed intervention. Histopathological examination suggested age-related degeneration rather than congenital or inflammatory etiology.

**Take-Home Messages:**

This is the first reported case of a noncoronary cusp SOVA rupturing into 2 left ventricular outflow tract fistulas. Careful hemodynamic assessment is crucial, as select patients may benefit from elective rather than urgent repair.

## Graphical Abstract

**Figure:**
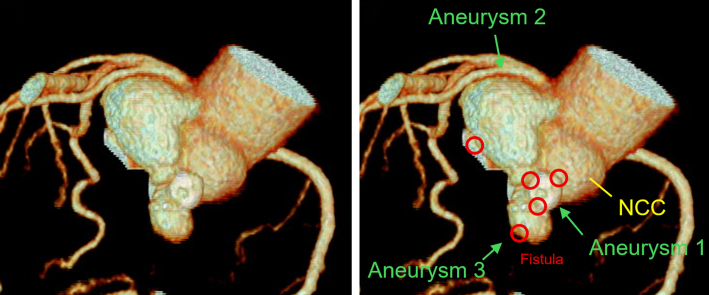

## History of Presentation

A 73-year-old woman was found to have ST-segment depression in leads V5 and V6 on an electrocardiogram obtained at her primary care clinic. Subsequent transthoracic echocardiography (TTE) identified a structure adjacent to the left atrium and moderate aortic regurgitation (AR), prompting referral to our institution.

## Past Medical History

The patient had a history of gastrointestinal AA amyloidosis and was treated 11 years prior with high-dose prednisolone and tocilizumab, chronic kidney disease, and hypertension.

## Differential Diagnosis

The differential diagnosis for the cause of this ruptured sinus of Valsalva aneurysm (SOVA) included: 1) age-related changes; 2) infective endocarditis; and 3) inflammatory conditions.

## Investigations

**Figure 1:**
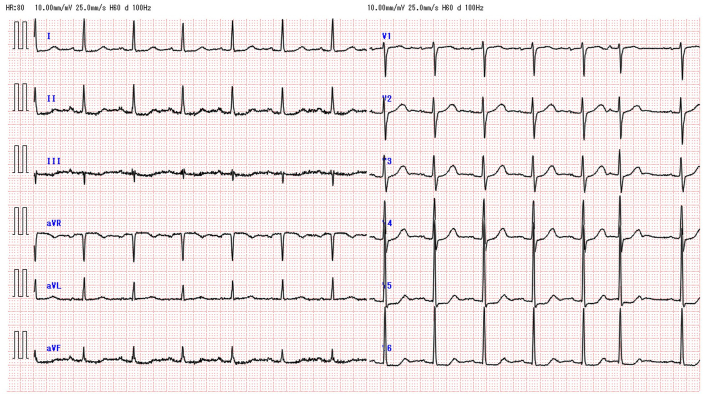
Electrocardiogram at the Outpatient Visit Electrocardiogram showed ST-segment depression in leads V5 and V6.

**Figure 2:**
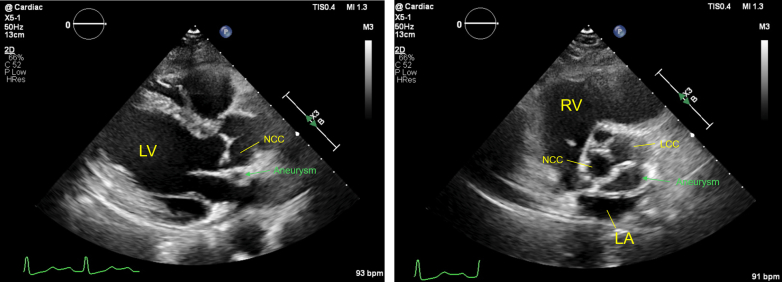
Transthoracic Echocardiography at the Outpatient Visit Transthoracic echocardiography revealed a membranous structure near the sinus of Valsalva on the side of the LCC and NCC. LA = left atria; LCC = left coronary cusp; LV = left ventricle; NCC = noncoronary cusp; RV = right ventricle.

**Figure 3:**
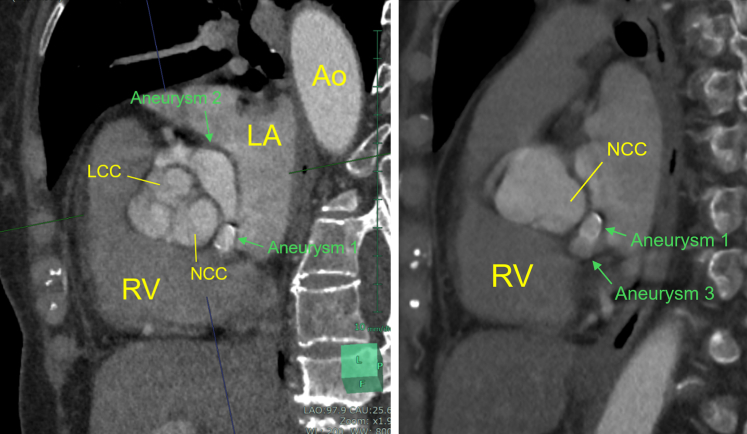
Preoperative Contrast-Enhanced CT The first aneurysm was a 10-mm-diameter aneurysm adjacent to the NCC (Aneurysm 1). The second aneurysm measured 14 mm in diameter and extended toward the LCC (Aneurysm 2). The third aneurysm measured 10 mm in diameter, was continuous with the first aneurysm, and extended toward the left ventricular outflow tract (Aneurysm 3). Ao = aorta; LA = left atria; LCC = left coronary cusp; LV = left ventricle; NCC = noncoronary cusp; RV = right ventricle.

**Figure 4:**
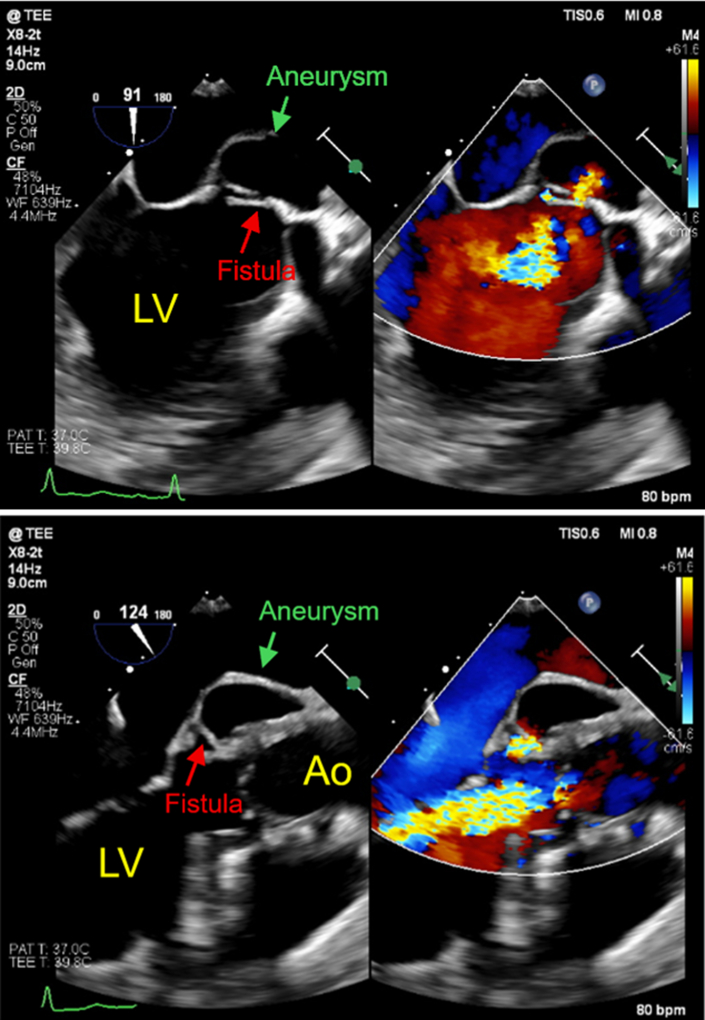
Preoperative Transesophageal Echocardiography Transesophageal echocardiography revealed a systolic jet from the aorta entering the NCC-adjacent aneurysm, which communicated with both the LVOT-side and NCC-side aneurysms via separate 3-mm-diameter channels. During diastole, a jet from the LCC-side aneurysm into the LVOT was observed. Moderate AR was noted.

**Figure 5:**
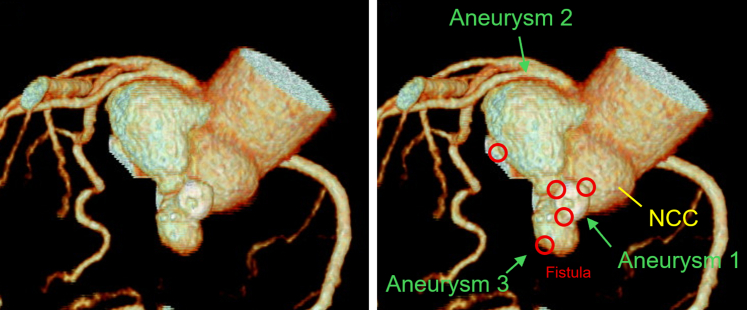
The Spatial Relationship Between the Aneurysm and the Fistula on 3D CT Reconstruction Systolic flow from the aorta entered the NCC-side aneurysm through a 3-mm-diameter fistulous connection. This aneurysm subsequently communicated via 3-mm-diameter fistulas with both the left ventricular outflow tract (LVOT)-side 10-mm-diameter aneurysm and the LCC-side 14-mm-diameter aneurysm, each of which also maintained communication with the LVOT.

The patient was asymptomatic, stood 145 cm tall, and had a brain natriuretic peptide level of 43.4 pg/mL. The electrocardiogram at the outpatient visit showed ST-segment depression in leads V5 and V6 (Figure 1). TTE revealed a membranous structure near the sinus of valsalva on the side of the left coronary cusp (LCC) and noncoronary cusp (NCC). The left ventricular end-diastolic/systolic diameters were 48/31 mm, the left ventricular posterior wall/interventricular septum thicknesses were 9/9 mm, and the left ventricular ejection fraction was 65%. The vena contracta measured 5.4 mm, indicating moderate AR (Figure 2). On 2D/3D cardiac CT at the coronary artery level, 3 saccular aneurysms surrounding the sinus of Valsalva were identified (Figure 3, Video 1, Video 2, Video 3). The first aneurysm was a 10-mm-diameter aneurysm adjacent to the NCC. The second aneurysm measured 14 mm in diameter and extended toward the LCC. The third aneurysm measured 10 mm in diameter, was continuous with the first aneurysm, and extended toward the left ventricular outflow tract (LVOT). Transesophageal echocardiography (TEE) revealed a systolic jet from the aorta entering the NCC-adjacent aneurysm, which communicated with both the LVOT-side and NCC-side aneurysms via separate 3-mm-diameter channels. During diastole, a jet from the LCC-side aneurysm into the LVOT was observed. Moderate AR was noted (Figure 4). These findings were further visualized using 3D CT reconstruction (Figure 5). Systolic flow from the aorta entered the NCC-side aneurysm through a 3-mm-diameter fistulous connection. This aneurysm subsequently communicated via 3-mm-diameter fistulas with both the LVOT-side 10-mm-diameter aneurysm and the LCC-side 14-mm-diameter aneurysm, each of which also maintained communication with the LVOT.

After multidisciplinary discussion between the departments of cardiology and cardiovascular surgery, it was determined that elective surgery was deemed feasible. The rationale for this treatment strategy is discussed in the Discussion section. Regular follow-up echocardiography in the outpatient setting demonstrated no evidence of rapid aneurysmal enlargement.

## Management

Elective surgery was performed 4 months after presentation.

**Figure 6:**
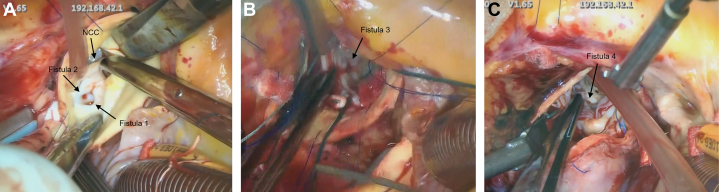
Intraoperative Images (A) A fistula (Fistula 1) to a sinus of Valsalva aneurysm was observed at the NCC, and a fistula (Fistula 2) to a saccular aneurysm on the LCC side was noted. (B) A fistula to the aneurysm (Fistula 3) was observed in the LVOT. (C) After aortic valve replacement, a fistula to the aneurysm (Fistula 4) was noted in the LVOT.

The surgical approach commenced with a median sternotomy, followed by the establishment of cardiopulmonary bypass through ascending aortic cannulation and right atrial cannulation. Cardioplegic arrest was subsequently achieved using a combination of retrograde and selective coronary perfusion. Upon aortotomy, inspection of the sinuses of Valsalva revealed an aneurysmal ostium located in the NCC (Figure 6A), where the NCC leaflet was significantly shortened. All 3 aortic leaflets were excised. The aneurysmal ostium in the NCC sinus was primarily closed using a Hemashield patch. In addition, an inflow site leading to the aneurysm was observed in the LVOT beneath the LCC (Figure 6B), which was closed with sutures.

For aortic valve replacement, a 19-mm Epic bioprosthetic valve was selected and secured to the aortic annulus using everting mattress sutures at the commissures and single interrupted sutures in the intercommissural spaces. The ascending aorta was then closed, with the suture line externally reinforced using autologous pericardium and Teflon felt. Following removal of the aortic cross-clamp, TEE indicated a residual communication between the LVOT and the aneurysm, necessitating re-clamping of the aorta and re-induction of cardioplegia. Inspection of the LVOT interior confirmed a second inflow site (Figure 6C), which was closed with sutures, and the aortic transection site was re-sutured.

Spontaneous cardiac rhythm resumed promptly, and the patient was weaned smoothly from cardiopulmonary bypass. After achieving meticulous hemostasis and thorough washout, the patient was transferred to the intensive care unit with stable hemodynamics.

**Figure 7:**
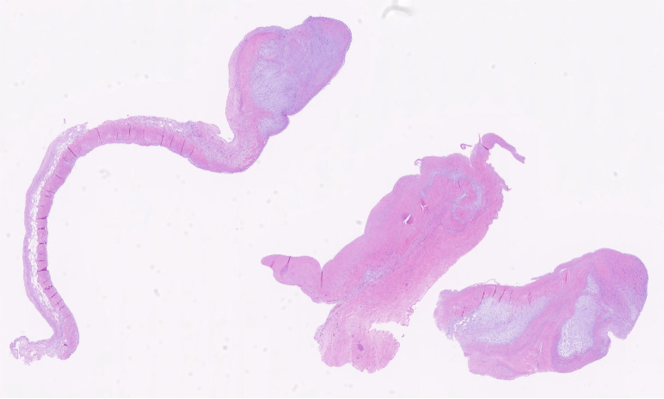
Biopsy of the Aortic Valve Cusp Pathological analysis of the aortic valve showed myxoid degeneration and mild hyalinization.

Pathological analysis of the aortic valve showed myxoid degeneration and mild hyalinization (Figure 7). In consideration of the risk of cardiac tamponade caused by leakage due to anastomotic dehiscence, a biopsy of the aneurysmal wall was not performed.

## Outcome and Follow-Up

The postoperative course was uneventful, and she was discharged on postoperative day 20. At 2-month follow-up, she remained asymptomatic. Due to worsening renal function (creatinine 1.45 mg/dL), a noncontrast CT scan was performed, which showed no recurrence of the SOVA (Video 4). She continues under regular medical observation.

## Discussion

Ruptured SOVAs commonly form fistulous tracts to adjacent cardiac chambers. While approximately 76% of cases involve ruptured congenital SOVAs, typically diagnosed at an average age of 30.8 years, the communication between the aorta and a cardiac chamber usually leads to progressive heart failure.^1^

The destination of the fistulous tract generally reflects the anatomical relationship of the sinus. While NCC-origin fistulas often connect to the right heart or interatrial septum,^2^ a 2023 systematic review by Ayati et al found that 97.7% of SOVA ruptures involved communication with the right heart, and only 1.7% involved the left heart.^3^ To our knowledge, there have been no previous reports of 2 simultaneous LVOT fistulas originating from an NCC-origin SOVA.

The ST-segment depression observed in leads V5 and V6 was not indicative of amyloidosis but was instead attributed to volume overload resulting from AR and the left-to-left (L-L) shunt. Since TTE at age 62 showed no evidence of an aneurysm, a congenital etiology seems less likely. Furthermore, pathological examination of the aortic valve revealed age-related changes, with no signs of connective tissue or inflammatory disease.

Potential causes of secondary SOVA include infectious, and inflammatory origins. In this patient, infectious etiologies—including *Staphylococcus* species, syphilis, and tuberculosis—were also considered unlikely. There were no signs of systemic infection or elevated inflammatory markers, and both the *Treponema pallidum* and rapid plasma reagin tests were negative. Furthermore, the patient exhibited no clinical features suggestive of tuberculosis, such as cough or hemoptysis, and imaging revealed no cavitary lesions. An inflammatory etiology, such as Behçet's disease–associated aortitis, was also considered. However, according to the International Criteria for Behçet's Disease, the patient scored only 1-2 points (vascular involvement present; pathergy test not performed), which does not meet the diagnostic threshold for Behçet's disease.^4^ Takayasu arteritis was another possible etiology; however, none of the diagnostic criteria proposed by the American College of Rheumatology (1990) were fulfilled, making this diagnosis unlikely.^5^ Given the presence of calcification in the aneurysmal wall of the sinus of Valsalva, the aneurysm in this case is most likely attributable to age-related degenerative changes.

**Figure 8:**
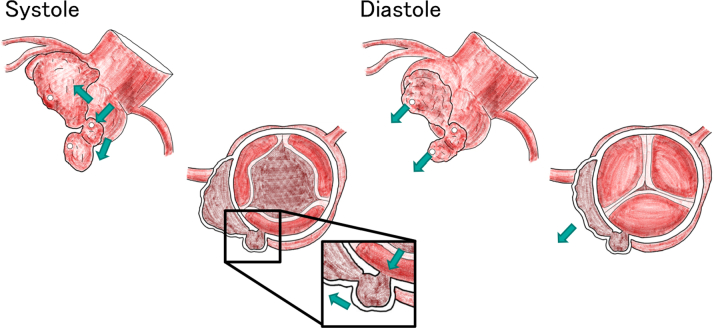
Blood Flow Within the Left-to-Left Shunt During Systole and Diastole During systole, blood flows from the aorta into the saccular aneurysm originating from the NCC, filling the LVOT-side and LCC-side aneurysms through the fistulas. During diastole, blood drains from the LVOT- and LCC-side aneurysms into the LVOT, resulting in decompression of the aneurysmal sacs. (Open circles: fistulas; green arrows: blood flow.)

The mechanisms that contributed to the decreased risk of aneurysmal rupture in this case are described below. During systole, blood flowed from the aorta into the saccular aneurysm originating from the NCC, and through the fistulas, both the LVOT-side and LCC-side aneurysms were filled. During diastole, blood drained from the LVOT- and LCC-side aneurysms into the LVOT, thereby decompressing the aneurysmal sacs. In addition, the small diameter (3 mm) of the connecting orifices between the aneurysms likely prevented direct systolic pressure transmission to the aneurysmal walls, further contributing to pressure reduction within the sacs (Figure 8).

Finally, we discuss the surgical indications. Because SOVA is rare, its surgical indications have not been clearly defined in existing guidelines. Therefore, the management strategy is generally extrapolated from the thoracic aortic aneurysm guidelines.^6^ According to the 2020 American College of Cardiology/American Heart Association Guidelines for Aortic Disease, emergency surgery is recommended in cases of ruptured aneurysm.^7^ However, in this patient, several factors supported an elective approach: the absence of genetic aortopathy such as Marfan syndrome, maintenance of systolic blood pressure between 100 and 120 mm Hg, aneurysmal diameter below 5.5 cm, no enlargement on TEE at 1-month follow-up, and a unique hemodynamic pattern that reduced the risk of open rupture through L-L shunt. This L-L shunt effectively prevented acute heart failure, which typically occurs with left-to-right shunts, allowing the patient to remain asymptomatic and hemodynamically stable.

According to the 2020 American College of Cardiology/American Heart Association and 2021 European Society of Cardiology/European Association for Cardio-Thoracic Surgery guidelines for the management of valvular heart disease, aortic valve replacement is considered reasonable (class IIa) in patients with moderate AR who are undergoing cardiac or aortic surgery for other indications.^7^^,^^8^ In this case, surgical repair of the ruptured SOVA required direct intervention at the aortic root; therefore, concomitant aortic valve replacement was performed in accordance with these recommendations.

## Conclusions

To the best of our knowledge, this is the first reported case of a SOVA originating from the NCC that ruptured and extended to form 2 simultaneous fistulous tracts into the LVOT. The formation of an L-L shunt between the aorta and the LVOT prevented the progressive heart failure typically associated with L-R shunts. This outcome was likely due to favorable hemodynamic conditions that facilitated sac decompression and pressure relief, thereby preventing acute rupture and permitting elective surgical intervention. Given the rarity of such presentations, further case accumulation is necessary to establish standardized therapeutic guidelines for similar SOVA management.

## Funding Support and Author Disclosures

The authors have reported that they have no relationships relevant to the contents of this paper to disclose.

**Take-Home Messages**

• Ruptured sinus of Valsalva aneurysm typically create right-sided shunts; however, this first reported case with 2 left ventricular outflow tract fistulas demonstrates that unique hemodynamic conditions can stabilize the patient.
• Detailed imaging and hemodynamic assessment may allow elective rather than urgent surgical intervention in selected cases.

## Appendix

Video 1 Preoperative Noncontrast CT Noncontrast CT demonstrated calcification in the wall of the sinus of Valsalva aneurysm adjacent to the noncoronary cusp. No pericardial effusion was observed.

Video 2 Preoperative Delayed-Phase Contrast-Enhanced CT The first aneurysm was a 10-mm-diameter aneurysm adjacent to the noncoronary cusp. The second aneurysm measured 10 mm in diameter and extended toward the left coronary cusp. The third aneurysm measured 14 mm in diameter, was continuous with the first aneurysm, and extended toward the left ventricular outflow tract.

Video 3 Preoperative 3D CT Reconstruction The morphology of the aneurysms was reconstructed using 3D CT. No coronary artery stenosis due to aneurysmal compression was observed.

Video 4 Postoperative Noncontrast CT at 2 Months Postoperatively There was no evidence of recurrence of the Valsalva sinus aneurysm.
